# Association between Gastroenterological Malignancy and Diabetes Mellitus and Anti-Diabetic Therapy: A Nationwide, Population-Based Cohort Study

**DOI:** 10.1371/journal.pone.0125421

**Published:** 2015-05-15

**Authors:** Chien-Ming Lin, Hui-Ling Huang, Fang-Ying Chu, Hueng-Chuen Fan, Hung-An Chen, Der-Ming Chu, Li-Wei Wu, Chung-Ching Wang, Wei-Liang Chen, Shih-Hua Lin, Shinn-Ying Ho

**Affiliations:** 1 Department of Pediatrics, Tri-Service General Hospital, National Defense Medical Center, Taipei, Taiwan; 2 Graduate Institute of Medical Sciences, National Defense Medical Center, Taipei, Taiwan; 3 Institute of Bioinformatics and Systems Biology, National Chiao Tung University, Hsinchu, Taiwan; 4 Department of Biological Science and Technology, National Chiao Tung University, Hsinchu, Taiwan; 5 Department of Family and Community Medicine, Tri-Service General Hospital, National Defense Medical Center, Taipei, Taiwan; 6 Division of Nephrology, Department of Medicine, Tri-Service General Hospital, National Defense Medical Center, Taipei, Taiwan; Kagoshima University Graduate School of Medical and Dental Sciences, JAPAN

## Abstract

**Background:**

The relationship between diabetes mellitus (DM) and cancer incidence has been evaluated in limited kinds of cancer. The effect of anti-diabetic therapy (ADT) on carcinogenesis among diabetic patients is also unclear.

**Materials and Methods:**

Using population-based representative insurance claims data in Taiwan, 36,270 DM patients and 145,080 comparison subjects without DM were identified from claims from 2005 to 2010. The association between the top ten leading causes of cancer-related death in Taiwan and DM was evaluated. Whether ADT altered the risk of developing cancer was also investigated.

**Results:**

Incidence of cancer at any site was significantly higher in patients with DM than in those without (*p*<0.001). The risk of carcinogenesis imparted by DM was greatest in gastroenterological malignancies (liver, pancreas, and colorectal cancer) as well as lung, breast and oral cancer (*p*<0.001). Among the oral types of ADT, metformin decreased the risk of lung and liver cancer, but had less effect on reducing the risk of colorectal cancer. α-glucosidase inhibitor decreased the risk of developing liver, colorectal, and breast cancer. Apart from intermediate-acting insulin, rapid-acting, long-acting, and combination insulin treatment significantly reduced the overall cancer risk among all DM patients. In subgroup analysis, long-acting insulin treatment significantly decreased the risk of lung, liver, and colorectal cancer.

**Conclusion:**

Our results supported the notion that pre-existing DM increases the incidence of gastroenterological cancer. ADT, especially metformin, α-glucosidase inhibitor, and long-acting insulin treatment, may protect patients with DM against these malignancies. It is crucial that oncologists should closely collaborate with endocrinologists to provide an optimal cancer-specific therapy and diabetic treatment to patients simultaneously with cancer and DM.

## Introduction

Although Diabetes mellitus (DM) and cancer are common diseases, their impacts on health care are tremendous. In Taiwan, epidemiologic studies have found that the prevalence of cancer and DM significantly increased from 2000 to 2009 [1]. Moreover, cancer and DM was the first and fifth leading causes of death in 2012, respectively [2]. These diseases attract public concerns because they are not only medical and health issues but also social and financial burdens globally. Therefore, health authorities try their best to find a good way to prevent and treat these annoying diseases.

Epidemiologic evidence suggests that cancer incidence is associated with DM itself, as well as certain diabetes risk factors and diabetes treatments [3,4]. Mechanistically, hyperglycemia may cause hyperinsulinemia, providing growth signals to positively stimulate the expansion of cancer [5–8]. Additionally, hyperglycemia may provide excessive energy sources to facilitate the growth of cancer [9–10]. Controversially, an *in vivo* model did not support that hyperglycemia could enhance neoplastic growth [11]. This discrepancy needs a large clinical data to clarify whether cancers are common in people with DM than in those without. If so, the role of ADT, which significantly influences the level of blood glucose, in the cancer incidence should be investigated as well.

In considering the complexity of the association between cancer, diabetes, and ADT, we conducted a nationwide, population-based cohort study to clarify the role of diabetes in the risk of developing cancers, taking advantage of a large-size data set available from the National Health Insurance (NHI) program in Taiwan. Whether the risk of cancer is reduced with the presence of DM therapies, including oral hypoglycemia agents and insulin injection, was also investigated.

## Materials and Methods

### Data sources

A single-player, compulsory NHI Program was launched by the Taiwanese government in March 1995. It provided coverage for 96% of the total population of Taiwan (23 million) in 2000 and 98% in 2005 [12]. The NHI Research Database (NHIRD), which is derived from NHI system, is set up for research purposes. The high coverage rate of the NHI Program makes the NHIRD the best national indicator of health issues. These data contain patients’ gender, age, medical expenditures, International Classification of Diseases, 9^th^ Revision, Clinical Modification (ICD-9-CM) codes, and types of prescribed medications, with the exceptions of clinical laboratory data and dose of medications.

### Cohort formation

The Longitudinal Health Insurance Database 2005 (LHID2005) contains all the original claims data of 1,000,000 beneficiaries, randomly sampled from the year 2005 Registry for Beneficiaries of the NHIRD. There are no significant differences in gender or age distribution or in the average insured payroll-related amounts between the patients in the LHID2005 and the original NHIRD. We studied the LHID2005 with antecedent data from 1^st^ January 2002, which allowed us to exclude people with prevalent DM during the period to 31^st^ December 2004. To obtain incident DM subjects, we formed a cohort of participants who were 20 years or older and were DM and cancer-free on 1^st^ January 2005 and non-use of DM medication from 2002–2004. This study was approved by the NHIRD research committee and institutional review board of Tri-service General Hospital. Since all identifying personal information was stripped from the secondary files before analysis, the review board waived the requirement for written informed consent from the patients involved.

### DM ascertainment, index date, and selecting controls

DM patients were defined as those who had at least one admission code or three or more outpatient codes for diabetes within one year during 2005–2010, and who were followed up for at least six months (*n* = 36,270). DM was classified into type 1 DM (T1DM, ICD 250.x1, 250.x3) (n = 1,447) and type 2 DM (T2DM, ICD 250.x0, 250.x2) (n = 34,823). The index date was the date the patients were first diagnosed with DM. The index date for the randomly-selected control subjects (non-diabetes, *n* = 145,080), corresponded to that of the DM study patients (*n* = 36,270), with the same gender-and-age (born in the same year) and at the ratio of case numbers 4:1. The selected control subjects were required to have been followed for at least 6 months and cancer-free during the first year after the index date, in the same way as the DM study patients. The two groups were followed up until the end of 2010 or the occurrence of cancer.

### Cancer event ascertainment and cancer incidence density

To investigate the relationship between the exposure (ADT) and outcome (cancer), we defined that incident cancer cases were only valid if they occurred at least one year after the index date until 31st December 2010. We considered only the first cancer in the second year or beyond and when the cancer diagnosis was recorded a second time within any year. To be cancer-free, there would be no record of cancer at any time after the index date. Otherwise, cancer status was regarded as uncertain and the subject deleted from the study. Cancers studied were the top ten leading causes of cancer-related death in Taiwan, including lung (ICD 162), liver (ICD 155, 156), colorectal (ICD 153, 154), breast (ICD 174), oral (ICD 140–141,143–146,148–149), stomach (ICD 151), prostate (ICD 185), pancreatic (ICD 157), esophageal (ICD 150) and cervix (ICD 179, 180) cancer [13].

Cancer incidence density (CID) was calculated as the number of incident cancer events divided by 10,000 person-years at risk followed (years after the index date until the first cancer diagnosis before the end of 2010 or, for non-cancer subjects, date of withdrawal from NHI or the end of 2010).

### Anti-diabetic therapy (ADT)

Antidiabetic agents were classified using the Anatomic Therapeutic Chemical (ATC) code. Since the NHI Program allows clinicians to use refillable prescriptions for chronic illness patients who need the same prescription medication, for example, getting three months’ medication per visit, ADT users were defined as those who had at least two ATC codes for antidiabetic drugs within six months after the index date. The duration of ADT treatment was defined as the time from the date of the first prescription record to 31^st^ December 2010 or until the occurrence of cancer. The oral ADTs were categorized into five groups: metformin (ATC code, A10BA), sulfonylureas (ATC code, A10BB), meglitinides (ATC code, A10BX), thiazolidinediones (TZDs) (ATC code, A10BG) and α-glucosidase inhibitor (ATC code, A10BF). The effect of oral ADTs on cancer risk, including lung, liver, colorectal, breast, oral and pancreatic cancer, was evaluated only for patients with T2DM. Moreover, insulin injection therapy was classified as rapid-acting (ATC code, A10AB), intermediate-acting (ATC code, A10AC), long-acting (ATC code, A10AE), and combination (ATC code, A10AD), to evaluate its effect on tumor development among the entire DM study cohort (n = 36,270).

### Statistical analysis

Baseline characteristics of subjects with and without DM were summarized and reported. For the comparison of cancer incidence in DM patients with and without ADT treatment for cancers, the hazard ratios (HRs) were estimated by Cox proportional-hazards models. In these models, the time variable was the interval between the index date and the date of cancer ascertainment, or date of withdrawal from NHI, or December 31, 2010. The potential covariates included sex, age, hypertension (ICD 401–405), dyslipidemia (ICD 272), obesity (ICD 278), gout (ICD 274), hepatitis B (ICD 070.2, 070.3), hepatitis C (ICD 070.4, 070.5, 070.7), liver cirrhosis (ICD 571), and duration of ADT exposure. These covariates were included in the models as categorical variables. All analyses were performed using SAS software version 9.1 (SAS Institute, Cary, NC), and Microsoft SQL Server 2008 software was used for data management. The level of statistical significance was set at a two-sided *P*<0.05.

## Results

The baseline characteristics of subjects with and without DM, such as age group, sex, urbanization, income and comorbidity, are shown in Table 1. Compared with the age-and sex-matched controls, the patients with DM were more likely to have the comorbidities of hypertension (54.3% vs 30.9%), dyslipidemia (57.1% vs 21.9%), obesity (1.9% vs 0.6%), gout (25.8% vs 12.9%), hepatitis B (6.4% vs 3.6%), hepatitis C (3.8% vs 1.8%) and liver cirrhosis (3% vs 1.3%) (all were p<0.001).

**Table 1:** Baseline characteristics of subjects with and without DM.

	Descriptor	Total no. of cases(n = 181350)	DM cases(n = 36270)		1:4 matched non-DM(n = 145080)		P value
		No.	No.	%	No.	%	
**Age (years)**	20–39	30125	6025	16.6	24100	16.6	1
	40–59	85335	17067	47.1	68268	47.1	
	≥60	65890	13178	36.3	52712	36.3	
	Mean(±SD)	53.13±15.17	54.26±14.76		52.57±15.33		
**Sex**	Female	89480	17896	49.3	71584	49.3	1
	Male	91870	18374	50.7	73496	50.7	
**Urbanization**	Provinces	47030	9324	25.7	37706	26	0.16
	Counties	14076	2832	7.8	11244	7.8	
	Districts	45656	9290	25.6	36366	25.1	
	Urbanvillages	74588	14824	40.9	59764	41.2	
**Income**	<18000	79165	15806	43.6	63359	43.7	0.75
	18000–34999	76027	15264	42.1	60763	41.9	
	≥35000	26158	5200	14.3	20958	14.4	
**Comorbidity**	Hypertension	64449	19686	54.3	44763	30.9	<0.001
	Dyslipidemia	52424	20714	57.1	31710	21.9	<0.001
	Obesity	1604	698	1.9	906	0.6	<0.001
	Gout	28135	9356	25.8	18779	12.9	<0.001
	HepatitisB	7473	2320	6.4	5153	3.6	<0.001
	HepatitisC	3908	1369	3.8	2539	1.8	<0.001
	Livercirrhosis	2970	1101	3	1869	1.3	<0.001

The risk of cancer at any site was significantly higher in patients with DM than in those without DM after adjusting for sex, age and comorbidities (*p*<0.001) (Table 2). Except for esophageal cancer, patients with DM had a higher risk of gastroenterological malignancy (e.g., liver, colorectum, and pancreas), and lung, breast, oral, stomach, prostate, and cervix cancer after adjusting for sex, age and comorbidities. However, the incidence rate of cancer was not significantly different between T1DM and T2DM patients (4.12 vs. 4.10 per 100,000 person-years) (Table 3).

**Table 2:** The risk of cancer in subjects with and without DM.

Descriptor	Total no. of cases	DM cases (n = 36270)	1:4 matched non-DM(n = 145080)	HR (95%CI)	Adjusted HR^a^(95%CI)
Cancer site	No. of cancer	No. of cancer (%)	No. of cancer (%)		
Any cancer site	16263	3639(22.4)	12624(77.6)	1.16(1.13–1.20) ^†^	1.46(1.41–1.52)^‡^
Lung	2295	485(21.1)	1810(78.9)	1.07(0.97–1.85)	1.40(1.26–1.55)^‡^
Liver	2373	660(27.8)	1713(72.2)	1.55(1.41–1.69) ^‡^	1.62(1.47–1.78)^‡^
Colorectum	2698	602(22.3)	2096(77.7)	1.15(1.05–1.26) ^†^	1.50(1.36–1.65)^‡^
Breast	1135	231(20.4)	904(79.6)	1.02(0.89–1.18)	1.36(1.16–1.59)^‡^
Oral cavity	730	160(21.9)	570(78.1)	1.13(0.95–1.34)	1.54(1.27–1.85)^‡^
Stomach	680	130(19.1)	550(80.9)	0.95(0.78–1.15)	1.24(1.01–1.51)^†^
Prostate	1137	245(21.5)	892(78.5)	1.10(0.96–1.27)	1.30(1.12–1.50)^†^
Pancreas	324	98(30.2)	226(69.8)	1.74(1.37–2.20) ^‡^	1.96(1.52–2.53)^‡^
Esophagus	353	56(15.9)	297(84.1)	0.74(0.56–0.99)	0.93(0.69–1.26)
Cervix	505	96(19)	409(81)	0.94(0.76–1.18)	1.36(1.08–1.72)^†^

Abbreviations: HR, hazard ratio.

^†^p< 0.05 for comparison between subjects with DM and without DM.

^‡^p< 0.001 for comparison between subjects with DM and without DM.

^a^Sex, age, hypertension, dyslipidemia, obesity, gout, hepatitis B, hepatitis C, and liver cirrhosis were adjusted for the cancer risk analysis.

**Table 3:** The characteristics of patients with DM (n = 36270).

Descriptor	Total	Type 1 DM	Type 2 DM	*P* value
**No. of DM (n)**	36270	1447	34823	<0.05
**Any sites of cancer (n)**	3639	148	3491	0.172
HR (95%CI)	1.16(1.13–1.20)^†^ ^b^	1.18(1.01–1.39)^†^ ^c^	1.15(1.11–1.19)^‡^ ^d^	-
Adjusted HR^a^ (95%CI)	1.46(1.41–1.52)^‡^ ^b^	1.47(1.25–1.73)^‡^ ^c^	1.34(1.29–1.39)^‡^ ^d^	-
**Cancer incidence density**	4.11	4.12	4.10	0.578

Abbreviations: CI, confidence interval, hazard ratio.

^†^p< 0.05,

^‡^p< 0.001

^a^Sex, age, hypertension, dyslipidemia, obesity, gout, hepatitis B, hepatitis C, and liver cirrhosis were adjusted for the cancer risk analysis.

^b^The comparison between subjects with DM and without DM.

^c^The comparison between subjects with T1DM and without DM.

^d^The comparison between subjects with T2DM and without DM.

For T2DM patients, metformin treatment decreased the risk of lung (AHR = 0.62 (0.45–0.85), p<0.05) and liver (AHR = 0.64 (0.49–0.83), p<0.05) cancer, but the risk of colorectal cancer was less decreased (AHR = 0.74 (0.53–1.03), p = 0.075) (Table 4). The risk of developing lung cancer was reduced after treatment with sulfonylureas (AHR = 0.69 (0.49–0.95), p<0.05) and TZDs (AHR = 0.37 (0.21–0.65), p<0.05). TZDs therapy also decreased the risk of oral cancer (AHR = 0.37 (0.16–1.85), p<0.05). Patients treated with α-glucosidase inhibitor had a decreased risk of developing liver (AHR = 0.58 (0.42–0.80), p<0.05), colorectal (AHR = 0.64 (0.44–0.93), p<0.05), and breast (AHR = 0.50 (0.26–0.98), p<0.05) cancer. However, there was no significant effect on malignancy with meglitinides therapy.

**Table 4:** Effects of oral ADT on the risk of cancer among T2DM patients.

Anti-diabetic drugs	Lung	Liver	Colorectum	Breast	Oral cavity	Pancreas
**Metformin (mean follow-up, days)**	978	1048	1143	1311	1631	1392
No. of patients (with/without)^a^	150/55	208/77	162/47	59/14	61/18	37/9
Cancer incidence density (with/without)^a^	4.7/10.3	6.6/14.4	5.1/8.8	1.9/2.6	1.9/3.4	1.2/0.2
Adjusted HR (95% CI)^b^	0.62(0.45–0.85)†	0.64(0.49–0.83)†	0.74(0.53–1.03)	0.74(0.41–1.34)	0.67(0.39–1.15)	0(0–0)
**Sulfonylureas (mean follow-up, days)**	1448	1375	1465	1470	1580	1274
No. of patients (with/without)^a^	157/48	241/44	166/43	54/19	72/7	41/5
Cancer incidence density (with/without)^a^	5.1/7.8	7.8/7.1	5.4/7.0	1.8/3.1	2.3/1.1	1.3/0.8
Adjusted HR (95% CI)^b^	0.69(0.49–0.95)†	1.05(0.76–1.45)	0.79(0.56–1.10)	0.62(0.37–1.06)	1.82(0.83–4.00)	1.77(0.70–4.50)
**Meglitinides (mean follow-up, days)**	1464	1596	1814	1266	1968	1553
No. of patients (with/without)^a^	26/179	42/243	26/183	8/65	7/72	4/42
Cancer incidence density (with/without)^a^	4.1/5.8	6.7/7.9	4.1/6.0	1.3/2.1	1.1/2.6	0.6/1.4
Adjusted HRs (95% CI)^b^	0.73(0.48–1.10)	0.73(0.53–1.02)	0.70(0.46–1.06)	0.65(0.31–1.06)	0.47(0.23–1.02)	0.46(0.17–1.30)
**Thiazolidinediones (mean follow-up, days)**	1489	1620	1853	1789	2359	1791
No. of patients (with/without)^a^	13/192	39/246	26/183	9/64	6/73	6/40
Cancer incidence density (with/without)^a^	1.7/6.3	5.7/8.2	3.8/6.1	1.3/2.1	0.9/2.4	0.8/1.3
Adjusted HR (95% CI)^b^	0.37(0.21–0.65)†	0.85(0.60–1.19)	0.74(0.49–1.12)	0.66(0.33–1.33)	0.37(0.16–1.85)†	0.71(0.30–1.70)
**α-Glucosidase inhibitor (mean follow-up, days)**	1617	1724	1867	1413	2090	1298
No. of patients (with/without)^a^	36/169	44/241	33/176	10/63	12/67	7/39
Cancer incidence density (with/without)^a^	4.0/6.1	4.9/8.6	3.7/6.3	1.1/2.3	1.3/2.4	0.8/1.4
Adjusted HR (95% CI)^b^	0.75(0.52–1.07)	0.58(0.42–0.80)†	0.64(0.44–0.93)†	0.50(0.26–0.98)†	0.58(0.31–1.07)	0.59(0.26–1.32)

Abbreviations: ADT, anti-diabetic therapy; CI, confidence interval; HRs, hazard ratios.

Cancer incidence density: the number of incident cancer events per 10,000 person-years.

^†^p< 0.05 for comparison between T2DM patients treated with and without oral ADT.

^a^Data presented as the T2DM patients treated with and without oral ADT.

^b^Adjusted for sex, age, hypertension, dyslipidemia, obesity, gout, hepatitis B, hepatitis C, liver cirrhosis, and duration of ADT exposure which was treated as a time dependent variable.

With regard to insulin injection therapy among all DM patients, rapid-acting (AHR = 0.40 (0.27–0.60)), long-acting (AHR = 0.60 (0.54–0.77)), and combination insulin treatment (AHR = 0.71 (0.58–0.86)) significantly reduced the risk of developing cancer at any site (all p<0.001) (Table 5). Long-acting insulin therapy also decreased the risk of developing lung (AHR = 0.51 (0.31–0.86)), liver (AHR = 0.66 (0.47–0.93)), colorectal (AHR = 0.40 (0.24–0.69)), and prostate cancer (AHR = 0.12 (0.30–0.49)) (all p<0.05). Combination insulin decreased the risk of colorectal (AHR = 0.47 (0.25–0.88)) and prostate cancer (AHR = 0.19 (0.05–0.77) (all p<0.05).

**Table 5:** Adjusted hazard ratios (AHRs) of cancer in DM patients with and without insulin injection.

Variables	Rapid-acting(95%Cl)	P-value	Intermediate-acting(95%Cl)	P-value	Long-acting(95%Cl)	P-value	Pre-mixed(95%Cl)	P-value
**Any cancer sites**	0.40(0.27–0.60)	<0.001	0.89(0.57–1.38)	0.6	0.60(0.51–0.72)	<0.001	0.71(0.58–0.86)	<0.001
**Lung**	0.28(0.07–1.11)	0.07	0.36(0.05–2.55)	0.31	0.51(0.31–0.86)	<0.05	0.72(0.42–1.25)	0.25
**Liver**	0.66(0.36–1.21)	0.18	1.52(0.75–3.05)	0.24	0.66(0.47–0.93)	<0.05	0.82(0.56–1.21)	0.31
**Colorectum**	0(0–0)	0	0.87(0.28–2.72)	0.82	0.40(0.24–0.69)	<0.05	0.47(0.25–0.88)	<0.05
**Breast**	0(0–0)	0	0.92(0.13–6.60)	0.94	0.56(0.25–1.26)	0.16	0.46(0.15–1.44)	0.18
**Oral cavity**	0.88(0.28–2.76)	0.82	1.73(0.43–7.01)	0.44	1.23(0.69–2.18)	0.48	1.49(0.78–2.85)	0.23
**Stomach**	0.49(0.07–3.55)	0.48	0(0–0)	0	0.62(0.26–1.53)	0.3	1.03(0.42–2.53)	0.95
**Prostate**	0.47(0.12–1.89)	0.29	0(0–0)	0	0.12(0.30–0.49)	<0.05	0.19(0.05–0.77)	<0.05
**Pancreas**	1.14(0.28–4.65)	0.86	0(0–0)	0	0.65(0.24–1.77)	0.39	1.91(0.88–4.15)	0.1
**Esophagus**	0.95(0.13–6.88)	0.96	0(0–0)	0	1.10(0.39–3.06)	0.86	0.85(0.21–3.52)	0.83
**Cervix**	0(0–0)	0	2.15(0.30–15.46)	0.45	1.22(0.49–3.03)	0.67	0.78(0.19–3.20)	0.73

AHRs: Adjusted for sex, age, hypertension, dyslipidemia, obesity, gout, hepatitis B, hepatitis C, liver cirrhosis, and duration of insulin injection exposure which was treated as a time dependent variable.

## Discussion

The underlying pathophysiological mechanisms of DM related to carcinogenesis are poorly understood but are probably multifactorial, including hyperglycemia facilitating neoplastic proliferation [9], hyperinsulinemia promoting carcinogenesis through its effects on insulin and/or IGF-I receptors [5–8], and inflammatory cytokines secreted by adipose tissue enhancing malignant progression [10]. Since insulin is produced by pancreatic β-cells and then transported via the portal vein to the liver, long-term exposure of growth factor (insulin) may case the higher incidences of cancer in pancreas and liver [3]. Additionally, the hyperinsulinemic state of diabetes, slower bowel transit time, and elevated fecal bile acid concentrations may facilitate the growth of colorectal cancer [4]. Herein, DM may potentially at least link to pancreatic, liver, and colorectal cancers. In agreement with previous reports [3–4], our large study sample, consisting of 36,270 patients with DM and 145,080 non-DM controls, substantiated that patients with pre-existing DM have a greater tendency to have a higher incidence of gastroenterological cancer, such as liver, pancreatic, and colorectal cancer, indirectly indicating that the hyperinsulinemic milieu may impose a greater risk of cancer growth.

If DM is significantly related to the incidence rate of cancers, it will be of interest to identify which type of DM is relevant to the higher incidence rate of cancers. In this study, we discovered that T1DM and T2DM both are significantly associated with the higher incidence of cancers compared to subjects without DM. However, an *in vivo* animal model showed that hyperglycemia did not lead to increased neoplastic growth [11]. We speculated non-modifiable risk factors (e.g., age, sex, race or ethnicity), undetermined hormonal derangements, duration of diabetes, treatment strategy, length of follow-up, sample sizes, and statistical adjustment for confounding factors may all have contributed to the conflicting results.

If the level of blood glucose is associated with a higher incidence of cancers, the role of ADTs may be a threatening risk of carcinogenesis in DM patients. Therefore, it is important to clarify the connection between the increase of cancer incidence and the use of ADT in DM patients. However, the association between diabetes and cancer may partly be due to shared common predisposing risk factors such as aging, obesity, diet, and physical inactivity. That is why there has been so much inconsistent evidence reported in observational studies showing that some ADTs are associated with an increased risk and some with a reduced risk of cancer [3,14–16]. To precisely determine the independent effect of ADT on the risk of malignancy, possible associated confounders including sex, age, hypertension, dyslipidemia, obesity, gout, hepatitis B, hepatitis C and liver cirrhosis were adjusted simultaneously in the present study. In the end, we found oral ADTs are associated with a decreased risk of some common cancers; these include metformin for lung and liver cancer, sulfonylurea for lung cancer, TZDs for lung and oral cancer, and α-glucosidase inhibitor for liver, colorectal, and breast cancer.

Several studies suggested that the use of metformin is associated with a reduced risk of cancer [15–23] or cancer mortality [24]. In agreement with reducing the risk of liver cancer, as in previous studies [14–16,25], our present study also demonstrated that metformin therapy decreased lung cancer risk, but had less effect on colorectal cancer. Metformin-induced activation of AMP-activated protein kinase (AMPK) may lead to inhibition of cell proliferation, reduce colony formation, and cause partial cell cycle arrest in cancer cell lines [26–29]. Moreover, the insulin-lowering action of metformin may contribute to its anti-neoplastic activity in vivo studies [27,30,31]. Appropriate use of metformin not only offers an anti-hyperglycemia effect but also inhibits carcinogenesis in the gastrointestinal (GI) system for T2DM patients.

α-glucosidase inhibitor may protect patients with DM against liver and colorectal cancer similar to metformin. With α-glucosidase inhibitor therapy, postprandial hyperglycemia in the GI system can be controlled by inhibiting the terminal step of carbohydrate digestion at the brush border of the intestinal epithelium. This unique mechanism may provide a plausible answer to explain why α-glucosidase inhibitor, rather than other oral ADTs, decreases the risk of GI malignancy. Nevertheless, our study showed the risk of pancreatic cancer, categorized as GI malignancy, remained unchanged with the use of any kind of oral ADT. Because the risk of developing cancer may increase as the duration of DM increases [16], the high mortality and short survival time of pancreatic cancer patients might mask the effect of oral ADT on carcinogenesis. However, a larger-scale prospective study might be necessary to clarify this possibility.

Another concern regarding diabetes and malignancy involves insulin therapy. Subcutaneous injection of insulin results in significantly higher levels of circulating insulin in the systemic circulation than does endogenous insulin secretion, thereby possibly amplifying the links between hyperinsulinemia and cancer risk through excessive insulin binding to the IGF-I receptor. Epidemiological studies suggested a higher frequency of malignancy in insulin glargine-treated patients [32,33]. However, a six-year international clinical trial, a 12,537-patient cardiovascular outcomes trial, found there was not a higher frequency of malignancy in glargine-treated patients [34]. By contrast, we were surprised to find that rapid-acting, long-acting, and combination insulin treatment had a tendency to reduce the development of cancer at any site. Long-acting insulin therapy not only decreased the development of lung and prostate cancer but also reduced oncogenesis in GI malignancies like liver and colorectal cancer. We speculated that the use of insulin therapies to control a hyperglycemic environment might also play a pivotal role in protecting some diabetic patients from carcinogenesis rather than the adverse effect of excessive exogenous insulin on IGF-I receptor resulting in cancer. We suggest that using an individually adequate insulin injection, and avoiding over-insulinization, is the best therapeutic strategy for current practice. Prospective long-term studies considering insulin injection dosage, comorbid conditions and the genetic background of diabetic patients are needed to further evaluate the true effect of exogenous insulin on malignancy.

There were some methodological weaknesses and strengths to this study. Firstly, socioeconomic (e.g., educational level, occupation), environmental, and biological factors (levels of hormones) well-known causes in carcinogenesis [35], were not completely recored in the NHIRD. Secondly, details on the dose of ADTs were lacking in the NHIRD. Thirdly, databases between the NHIRD and “National Cancer Registry” can be traced and analysed, but the link between the NHIRD and the “National Registry of Deaths” is not available. Therefore, the association between non-cancer deaths and DM could not be evaluated. Even though some weaknesses existed in the present study, there is a probable completeness of ascertainment of the diagnoses of cancer and diabetes using the computerized data file for each individual from the NHIRD, which is population-based and highly representative, resulting in little possibility of recall and selection bias. Another strength of the study is that the effect of all kinds of ADTs on developing cancer among diabetic patients was assessed thoroughly in this article.

## Conclusions

Although DM and cancer share many common risk factors, our population-based retrospective cohort study demonstrated DM may potentiate gastroenterological carcinogenesis. Appropriate use of ADTs with metformin, α-glucosidase inhibitor and long-acting insulin might have a protective effect against the development of liver and colorectal cancer.
